# Inflammatory mediators and dual depression: Potential biomarkers in plasma of primary and substance-induced major depression in cocaine and alcohol use disorders

**DOI:** 10.1371/journal.pone.0213791

**Published:** 2019-03-14

**Authors:** Nuria García-Marchena, Marta Barrera, Joan Ignasi Mestre-Pintó, Pedro Araos, Antonia Serrano, Clara Pérez-Mañá, Esther Papaseit, Francina Fonseca, Juan Jesús Ruiz, Fernando Rodríguez de Fonseca, Magí Farré, Francisco Javier Pavón, Marta Torrens

**Affiliations:** 1 Unidad Gestión Clínica de Salud Mental, Instituto de Investigación Biomédica de Málaga (IBIMA), Hospital Regional Universitario de Málaga/Universidad de Málaga, Málaga, Spain; 2 Addiction Research Group, Hospital del Mar Medical Research Institute (IMIM), Barcelona, Spain; 3 Universitat Pompeu Fabra (UPF), Barcelona, Spain; 4 Departamento de Psicobiología y Metodología de las Ciencias del Comportamiento, Instituto de Investigación Biomédica de Málaga (IBIMA), Facultad de Psicología, Universidad de Málaga (UMA), Málaga, Spain; 5 Hospital Universitari Germans Trias i Pujol (IGTP), Badalona, Spain; 6 Universitat Autònoma de Barcelona (UAB), Barcelona, Spain; 7 Institut de Neuropsiquiatria i Addiccions (INAD), Barcelona, Spain; 8 Centro Provincial de Drogodependencias, Diputación Provincial de Málaga, Málaga, Spain; Shinshu University School of Medicine, JAPAN

## Abstract

Major depressive disorder (MDD) is the most prevalent comorbid mental disorder among people with substance use disorders. The MDD can be both primary and substance-induced and its accurate diagnosis represents a challenge for clinical practice and treatment response. Recent studies reported alterations in the circulating expression of inflammatory mediators in patients with psychiatric disorders, including those related to substance use. The aim of the study was to explore TNF-α, IL-1β, CXCL12, CCL2, CCL11 (eotaxin-1) and CX3CL1 (fractalkine) as potential biomarkers to identify comorbid MDD and to distinguish primary MDD from substance-induced MDD in patients with substance disorders. Patients diagnosed with cocaine (CUD, n = 64) or alcohol (AUD, n = 65) use disorders with/without MDD were recruited from outpatient treatment programs [CUD/non-MDD (n = 31); CUD/primary MDD (n = 18); CUD/cocaine-induced MDD (N = 15); AUD/non-MDD (n = 27); AUD/primary MDD (n = 16) and AUD/alcohol-induced MDD (n = 22)]. Sixty-two healthy subjects were also recruited as control group. Substance and mental disorders were assessed according to “Diagnostic and Statistical Manual of Mental Disorders, 4^th^ edition, text revision” (DSM-IV-TR) and a blood sample was collected for determinations in the plasma. The cocaine group showed lower TNF-α (*p*<0.05) and CCL11 (*p*<0.05), and higher IL-1β (*p*<0.01) concentrations than the control group. In contrast, the alcohol group showed higher IL-1β (*p*<0.01) and lower CXCL12 (*p*<0.01) concentrations than the control group. Regarding MDD, we only observed alterations in the cocaine group. Thus, CUD/MDD patients showed lower IL-1β (*p*<0.05), CXCL12 (*p*<0.05) and CCL11 (*p*<0.05), and higher CXC3CL1 (p<0.05) concentrations than CUD/non-MDD patients. Moreover, while CUD/primary MDD patients showed higher CCL11 (*p*<0.01) concentrations than both CUD/non-MDD and CUD/cocaine-induced MDD patients, CUD/cocaine-induced MDD patients showed lower CXCL12 (*p*<0.05) concentrations than CUD/non-MDD patients. Finally, a logistic regression model in the cocaine group identified CXCL12, CCL11 and sex to distinguish primary MDD from cocaine-induced MDD providing a high discriminatory power. The present data suggest an association between changes in inflammatory mediators and the diagnosis of primary and substance-induced MDD, namely in CUD patients.

## Introduction

Major depressive disorder (MDD) is the most prevalent comorbid mental condition in subjects with substance use disorders (SUD) and closely related to their poor prognosis. As expected, due to their marked clinical severity, these dual diagnosed patients also present considerable psychosocial acuteness and make greater use of health resources, including emergency services and psychiatric admissions [1–3]. The identification of MDD in substance users, however, is often complicated because of its inherent characteristics, that is to say, the acute and chronic effects related to substance consumption/withdrawal can mimic depression. Currently, diagnosis is syndromic and established with clinical criteria using “Diagnostic and Statistical Manual of Mental Disorders 4^th^ Edition Text Revision” (DSM-IV-TR), “DSM 5^th^ Edition” (DSM-5) [4–5] or “International Classification of Diseases and Related Health Problems 10^th^ Edition” (ICD-10) [6], the availability of specific biomarkers to facilitate the diagnosis of MDD in SUD are therefore required [7]. Among others, modulation of inflammatory mediators such as cytokines and chemokines has been recognized as a possible target. A growing body of literature has established that cytokines can play a critical role in the pathogenesis of both MDD [8–9] and SUD [10–13]. Preclinical and clinical studies in MDD and SUD have reported alterations in the immune system and dysregulation of the hypothalamic-pituitary-adrenal axis (HPA) [14–15]. These changes result in a chronic inflammatory response and a disruption of brain integrity and homeostasis as consequence of neuroinflammation [16]. In the brain, glial activation results in the release of cytokines such as tumor necrosis factor-alpha (TNF-α) and interleukin-1 beta (IL-1β), inflammatory mediators that influence the central immune system, modulate neural activity, and regulate the HPA axis [14, 17–18]. Chemokines, small chemoattractant proteins, also act as modulators in neuronal transmission and participate in the communication between glia and neurons [8, 19].

An increasing quantity of research has focused on the leading role of inflammatory mediators in the pathophysiology of a variety of psychiatric problems including bipolar [20] and post-traumatic stress disorders [21], schizophrenia [22], and MDD [23]. With respect to MDD, patients show increased serum levels of TNF-α and it has been reported that elevated serum levels of CCL11 are associated with suicidal ideations in such patients [24]. Moreover, there are studies suggesting that inflammatory markers have the potential to predict antidepressant treatment outcomes [25–26]. Regarding SUD, preclinical models of cocaine exposure have described that stromal derived factor-1/SDF-1 (CXCL12) participates in the modulation of cocaine-induced behavioral effects such as locomotion and stereotypes [27]. Previous studies in abstinent subjects with CUD found a positive correlation between plasma levels of IL-1β, fractalkine (CX3CL1), and CXCL12 with a number of DSM-IV-TR criteria for CUD, and with increased prevalence of comorbid psychiatric disorders relative to those users with no other psychiatric diagnosis [11]. With respect to alcohol, in preclinical models it has been reported to disrupt the cytokine profile during neuronal differentiation and influence adult neurogenesis, thus providing potential mechanisms to understand alcohol effects on cerebral development [28]. Other clinical studies have indicated that alcohol dependence is characterized by enhanced glial IL-1β in the cerebral cortex [29] and elevated chemokine monocyte chemotactic protein 1/MCP-1 (CCL2) in different brain regions [10]. Furthermore, a recent study in patients diagnosed with alcohol use disorders (AUD) showed lower plasma levels of CXCL12 and CX3CL1 than in controls. Additionally, AUD patients with comorbid depressive and/or anxiety disorders have been reported to have alterations in the plasma levels of the chemokine eotaxin-1 (CCL11) [30].

Because the peripheral alterations of inflammatory molecules may modulate and/or reflect neuroinflammatory events in the brain, certain cytokines and chemokines could be reliable candidates to detect MDD in SUD patients. Changes in peripheral inflammatory mediators might thus contribute to improving diagnosis, treatment strategy, and medical approach for these psychiatric patients [7, 31]. The aim of the present study is to explore whether relevant proinflammatory cytokines such as TNF-α, and IL-1β, and chemokines such as CXCL12, CCL2, CCL11, and CX3CL1 could be potential biomarkers for the detection of co-occurrence of MDD in patients with CUD and/or AUD, and to distinguish primary MDD from substance-induced MDD.

## Materials and methods

### Subjects

The present cross-sectional study was conducted in white Caucasian population, and included SUD [cocaine (CUD) or alcohol (AUD)] patients diagnosed with/without comorbid MDD, and healthy control subjects. Patients were recruited at the addiction treatment facilities of the *Instituto de Neuropsiquiatría y Adicciones-Parc Salut Mar* (Barcelona, Spain), *Hospital Universitario 12 de Octubre* (Madrid, Spain), and Centro Provincial de Drogodependencias (Málaga, Spain). Control participants were included from data bases of healthy subjects willing to participate in medical research projects at the *Unidad de Farmacología* del *IMIM-Hospital del Mar* Research Institute (Barcelona, Spain), *Hospital Universitario 12 de Octubre* (Madrid, Spain), and *Hospital Regional Universitario de Málaga* (Málaga, Spain).

Cocaine (n = 64) and alcohol (n = 65) groups were classified into six different subgroups: CUD with no MDD (CUD/non-MDD, n = 31), CUD with primary MDD (CUD/primary MDD, n = 18); CUD with cocaine-induced MDD (CUD/cocaine-induced MDD, n = 15), AUD with no MDD (AUD/non-MDD, n = 27), AUD with primary MDD (AUD/primary MDD, n = 16); AUD with alcohol-induced MDD (AUD/alcohol-induced MDD, n = 22).

All the subjects met the inclusion criteria, which were: both genders, age ≥ 18 years old up to 65 years of age and at least 4 weeks of abstinence in the case of subjects with SUD. Those patients who were diagnosed with MDD had to be in remission and “Hamilton Depression Rating Scale” (HDRS) scores had to be lower than 6. We considered 4 weeks of abstinence (SUD) and remission phase (MDD) because patients have to be as pathophysiologically stable as possible for clinical assessments and biochemical determinations. The exclusion criteria included: cognitive or language limitations that precluded evaluations and pregnant or breastfeeding women. SUD patients suffering from any psychiatric disorder in Axis I (DSM-IV-TR) [4] other than MDD and/or CUD/AUD (except from nicotine use disorder) were excluded. In the control group, participants with psychiatric disorders in Axis I (DSM-IV-TR), SUD (except nicotine use disorder) or family history of MDD were also excluded. Other excluding factors for all participants were: use of anti-inflammatory drugs or MAOI’s, personal history of cancer; major surgery within 6 months prior to the study; long-term inflammatory disease; any chronic illnesses that could interfere in the study such as cardiovascular, respiratory, renal, hepatic, endocrine, gastro-intestinal, hematological, neurological diseases; and infectious diseases (including HIV, hepatitis B, and hepatitis C). Clinical assessments and blood/urine analyses were performed to assure these participation criteria.

### Ethics statements

Written informed consents were obtained from each participant after a complete description of the study. All the participants had the opportunity to discuss any questions or issues. The study and protocols for recruitment were approved by the Ethics Committee of the *Hospital Regional Universitario de Málaga* (CP14/00173, CP14/00212 and PI13/02261) and by the Research Ethical Committee from the CEIC-*Parc de Salut Mar* (2012/4903/I; 2012/4751/I; 2009/3494/I) in accordance with the Ethical Principles for Medical Research Involving Human Subjects adopted in the Declaration of Helsinki by the World Medical Association (64^th^ WMA General Assembly, Fortaleza, Brazil, October 2013), Recommendation No. R (97) 5 of the Committee of Ministers to Member States on the Protection of Medical Data (1997), and Spanish data protection act (*Ley Orgánica* 15/1999 de *Protección de Datos*, *LOPD*). All collected data were given code numbers in order to maintain privacy and confidentiality.

### Clinical assessments

Substance and non-substance use disorders were diagnosed according to the DSM-IV-TR criteria [4] using the Spanish version of the “Psychiatric Research Interview for Substance and Mental Disorders” (PRISM) [4, 32]. PRISM is a semi-structured interview that has demonstrated good psychometric properties in terms of test-retest reliability, inter-rater reliability, and validity for primary MDD and substance-induced MDD, with kappa ranging from 0.66 and 0.75 [32–33]. All the interviews were performed by trained and experienced psychologists.

### Collection of plasma samples

Blood samples were obtained in the morning after fasting for 8–12 h by experienced nurses. Participants were comfortably seated in a chair for 5 min before the blood draw, and were asked whether they had allergies, phobias or a history of vaso-vagal response during previous injections or blood draws. The participants could be placed in the supine position by adjusting the chair (backrest) if necessary. After the blood draw, participants were invited for breakfast prior to the psychiatric interviews.

Venous blood was extracted into 10 mL K_2_ EDTA tubes (BD, Franklin Lakes, NJ, USA) and immediately processed to obtain plasma. Blood samples were centrifuged at 2,200 x g for 15 min (4°C) and individually assayed to detect infectious diseases by 3 commercial rapid tests for HIV, hepatitis B, and hepatitis C (Strasbourg, Cedex, France). Finally, plasma samples were individually characterized, registered, and stored at -80°C until further analyses.

### Multiplex immunoassays

Proinflammatory cytokines and chemokines were chosen based on previous studies regarding relevant inflammatory mediators in psychiatric disorders and SUD using a Luminex Platform for quantification [11, 13, 34]. A Bio-Plex Suspension Array System 200 (Bio-Rad Laboratories, Hercules, CA, USA) was used to quantify cytokine and chemokine concentrations in plasma with a ProcartaPlex Immunoassay panel and an appropriate Plasma Standard Diluent Kit (Invitrogen, ThermoFisher Scientific, Waltham, MA, USA). A human cytokine/chemokine 6-plex panel was used to simultaneously detect the following analytes: TNF-α, IL-1β, CXCL12; CCL2, CCL11, and CX3CL1. The measurements of these inflammatory mediators in plasma were performed following the manufacturer´s instructions [11]. Raw data (mean fluorescence intensity) were analyzed using the Bio-Plex Manager Software 4.1 (Bio-Rad laboratories, Hercules, CA, USA). Plasma concentrations were expressed as pg/mL. According to manufacturer’s specifications (ProcartaPlex Human Cytokine/Chemokine Simplex Kit), assay sensitivity and standard curve range for each analyte were as follows: 0.4 pg/mL and 8.54–35,000 pg/mL for TNF-α (catalog #: EPX01A-10223-901); 0.2 pg/mL and 2.44–10,000 pg/mL for IL-1β (catalog #: EPX01A-10224-901); 20.5 pg/mL and 17.1–70,000 pg/mL for CXCL12 (catalog #: EPX01A-12138-901); unknown and 37.5–23,300 pg/mL for CCL2 (catalog #: EPX01B-10281-901); 1.4 pg/mL and 0.61–2,500 pg/mL for CCL11 (catalog #: EPX01B-12120-901); and 0.5 pg/mL and 2.08–8,500 pg/mL for CX3CL1 (catalog #: EPX01A-12121-901). Inter-assay and intra-assay (samples in duplicate) coefficients of variation (%CV) were as follows: 7.1% and 6.5% for TNF-α; <5% and 8.9% for IL-1β; 7.8% and 9.0% for CCL11; 7.2% and 6.4% for CX3CL1; <5% and 9.8% for CCL2; and 5% and 10.7% for CXCL12.

### Statistical analysis

All data in the tables are expressed as number and percentage of subjects [n (%)] or mean and standard deviation [mean (SD)]. The significance of differences in categorical and normal continuous variables was determined using Fisher’s exact test (chi-square test) and Student’s *t*-test, respectively.

One-way analysis of covariance (ANCOVA) was performed to assess the effects of independent factors (i.e., grouping variables such as types of SUD, diagnosis of MDD, and types of MDD) on the plasma concentrations of cytokines and chemokines (dependent variables), controlling for additional independent variables and covariates [e.g., sex, age, body mass index (BMI), and antidepressant medication]. The *post hoc* tests for multiple comparisons were performed using Sidak’s correction test. Prior to performing one-way ANCOVA, logarithm (10)-transformation for dependent variables was used to ensure statistical assumptions for positive skewed distributions.

Binary logistic models were employed to distinguish between primary MDD and substance-induced MDD for SUD patients using full models that included cytokine and chemokine concentrations, sex, age, and BMI, and adjusted models (variables were chosen using a backward stepwise approach). Receiver operating characteristic (ROC) analysis was performed to determine the accuracy of these models comparing the area under the curve (AUC) and representative cut-off scores for the distinction between types of MDD, which included estimations of sensitivity and specificity.

Statistical analysis was carried out with the GraphPad Prism version 5.04 (GraphPad Software, San Diego, CA, USA), and IBM SPSS Statistical version 22 (IBM, Armonk, NY, USA). A *p*-value<0.05 was considered statistically significant.

## Results

### Sociodemographic and clinical characteristics of the sample

Table 1 shows the sociodemographic characteristics and depression-related variables of 191 participants in this cross-sectional study who were divided into 3 groups: cocaine, alcohol and control groups.

**Table 1:** Baseline sociodemographic and clinical characteristics of the study sample.

VARIABLES		CONTROL GROUP (n = 62)	COCAINE GROUP (CUD) (n = 64)			ALCOHOL GROUP (AUD) (n = 65)		
			Non-MDD (n = 31)	Primary MDD (n = 18)	Cocaine-Induced MDD (n = 15)	Non-MDD (n = 27)	Primary MDD (n = 16)	Alcohol-Induced MDD (n = 22)
**SOCIODEMOGRAPHIC VARIABLES**								
**Age** *[Mean (SD)]*	*Years*	34.82 (6.23)	36.52 (6.47)	40.89 (10.11)	35.67 (9.34)	46.78 (6.71) ^**aaa**^	47.31 (6.03) ^**aaa**^	46.64 (8.77) ^**aaa**^
**BMI** *[Mean (SD)]*	*Kg/m*^*2*^	24.89 (3.49)	24.64 (3.05)	26.69 (5.75)	26.16 (4.23)	25.63 (3.87)	27.68 (4.58) ^**a**^	25.23 (3.57)
**Sex** *[n (%)]*	Women	29 (46.8)	6 (19.4) ^**a**^	5 (27.8)	4 (26.7)	7 (25.9)	5 (31.3)	10 (45.5)
	Men	33 (53.2)	25 (80.6)	13 (72.2)	11 (73.3)	20 (74.1)	11 (68.8)	12 (54.5)
**Marital Status** *[n (%)]*	Single	28 (45.2)	12 (38.7)	6 (33.3) ^**aa**^	11 (73.3)	9 (33.3)	3 (18.8)	8 (36.4)
	Cohabiting	24 (38.7)	11 (35.5)	3 (16.7)	1 (6.7)	13 (48.1)	7 (43.8)	9 (40.9)
	Separated	6 (9.7)	8 (25.8)	9 (50.0)	3 (20.0)	5 (18.5)	5 (31.3)	4 (18.2)
	Widow/er	4 (6.5)	-	-	-	-	1 (6.3)	1 (4.5)
**Education** *[n (%)]*	Elementary	1 (1.6)	16 (51.6) ^**aaa**^	10 (55.6) ^**aaa**^	7 (46.7) ^**aaa**^	10 (37.0) ^**aaa**^	4 (25.0) ^**aaa**^	6 (27.3) ^**aaa**^
	Secondary	22 (35.5)	11 (35.5)	4 (22.2)	4 (26.7)	14 (51.9)	8 (50.0)	7 (31.8)
	University	39 (62.9)	4 (12.9)	4 (22.2)	4 (26.7)	3 (11.1)	4 (25.0)	9 (40.9)
**Occupation** *[n (%)]*	Employed	41 (66.1)	15 (48.4)	10 (55.6)	4 (26.7) ^**aa**^	12 (44.4)	6 (37.5)	9 (40.9)
	Unemployed	21 (33.9)	16 (51.6)	8 (44.4)	11 (73.3)	15 (55.6)	10 (62.5)	13 (59.1)
**DEPRESSION-RELATED VARIABLES**								
**Age of onset** *[Mean (SD)]*	*Years*	-	-	33.33 (11.30)	31.40 (8.63)	-	42.31 (7.67)	39.29 (9.21)
**Number of episodes** *[Mean (SD)]*		-	-	2.44 (1.89)	4.20 (6.47)	-	3.54 (5.13)	4.00 (5.66)
**Age at last episode** *[Mean (SD)]*	*Years*	-	-	38.61 (10.25)	31.50 (5.17) ^**c**^	-	47.23 (6.07)	46.64 (8.77)
**Remission** [*Mean (SD)]*	*Months*	-	-	15.00 (19.88)	29.56 (35.64)	-	6.38 (4.05)	14.24 (18.24)
**Current antidepressant treatment** *[n (%)]*		-	-	10 (55.6)	4 (26.7)	5 (18.5)	11 (68.8) ^**bb**^	9 (40.9)
**Family history of MDD** *[n (%)]*		-	5 (16.1)	9 (50.0)	4 (26.7)	15 (55.6)	9 (56.3)	12 (54.5)

^**(a)**^ *p*<0.05

^**(aa)**^ *p*<0.01 and

^**(aaa)**^ *p*<0.001 denote significant differences versus control group

^**(bb)**^ *p*<0.01 denotes significant differences versus non-MDD subgroup

^**(c)**^ *p*<0.05 denotes significant differences versus primary MDD subgroup

Abbreviations: AUD = alcohol use disorders; BMI = body mass index; CUD = cocaine use disorders MDD = major depressive disorders

The mean age of the cocaine group was 37.6 years, and 23.4% were women. The most common marital status was single, mainly in cocaine-induced MDD patients, with elementary education (51.6%). As compared with the control group, we observed significant differences in the percentage of women (CUD/non-MDD patients were 19% women; *p*<0.05), marital status (CUD/primary MDD patients were mainly separated; *p*<0.01), education (*p*<0.001), and occupation (CUD/cocaine-induced MDD patients were mainly unemployed; *p*<0.01). In the alcohol group, the mean age was 46.9 years, and 33.8% were women. In contrast, cohabiting was the most common marital status, and education at secondary or higher level reached 69.2%. The comparison with the control group revealed significant differences in the mean age (*p*<0.001), BMI (AUD/primary MDD had higher BMI than controls; *p*<0.05), and education (*p*<0.001).

Regarding MDD-related variables in the cocaine group, the mean age of onset was 32.5 years and 3.2 episodes. However, there were significant differences in the age of the last episode when primary and cocaine-induced patients were compared (*p*<0.05). In the alcohol group, the mean age of onset was 40.6 years and 3.81 episodes. No differences were found in the age of the last episode in AUD patients. Finally, although remission and current treatment with antidepressants were apparently different as compared both primary and substance-induced MDD subgroups in the cocaine and alcohol groups, statistical analysis found no significant differences.

### Inflammatory mediators in relation to substance use disorders

The impact of SUD on the plasma concentrations of proinflammatory cytokines and chemokines was investigated using a one-way ANCOVA [cocaine group (CUD/non-MDD patients), alcohol group (AUD/non-MDD patients), and control group], and controlling for sex, age, and BMI. Plasma concentrations and statistical analysis of inflammatory mediators according to SUD are described in Table 2.

**Table 2:** Plasma concentrations of inflammatory mediators in participants grouped according to the type of substance use disorder.

VARIABLE	CONTROL GROUP (n = 62)	COCAINE GROUP (CUD) Non-MDD (n = 31)	ALCOHOL GROUP (AUD) Non-MDD (n = 27)	ANCOVA (Statistics)^(1)^		
				F-value	df	*p*-value
	Mean ± SD	Mean ± SD	Mean ± SD			
**TNF-α** (pg/mL)	11.32±4.91	8.00±5.11 ^**a**^	10.27±5.97	4.540	2, 113	**0.013**
**IL-1β** (pg/mL)	0.724±0.76	1.346±0.87 ^**aa**^	1.764±1.13 ^**aa**^	7.891	2,103	**0.001**
**CXCL12** (pg/mL)	287.2±44.53	287.9±60.34	240.1±77.69 ^**aa**^^,^ ^**b**^	5.662	2,114	**0.005**
**CCL2** (pg/mL)	35.29±16.37	38.70±9.303	39.01±16.28	2.198	2, 114	0.116
**CCL11** (pg/mL)	148.5±61.24	126.5±54.00 ^**a**^	128.8±37.10	3.737	2, 114	**0.027**
**CX3CL1** (pg/mL)	2.606±1.37	1.987±1.67	1.839±1.09	1.886	2, 113	0.156

^**(1)**^ Statistical analysis was conducted on the logarithmic transformed values to ensure statistical assumptions. Data were analyzed by ANCOVA controlling for age, sex, and BMI

^**(a)**^ *p*<0.05 and

^**(aa)**^ *p*<0.01 denote significant differences versus control group after *post hoc* comparisons

^**(b)**^ *p*<0.05 denotes significant differences versus cocaine non-MDD group after *post hoc* comparisons

Missing participants: TNF-α (n = 1, control); IL-1β (n = 4, control; n = 7, CUD); CX3CL1 (n = 1, CUD)

Abbreviations: ANCOVA = analysis of covariance; AUD = alcohol use disorders; CUD = cocaine use disorders; df = degree of freedom; Non-MDD = non-major depressive disorders

One-way ANCOVA revealed a main effect of SUD on TNF-α (*p*<0.05) concentrations. While the cocaine group showed significantly lower TNF-α concentrations than the control (*p*<0.05), there were no differences in TNF-α concentrations between the alcohol group and the control. In the case of IL-1β, there was also a main effect of SUD (*p*<0.01) and both cocaine and alcohol groups reported higher IL-1β concentrations than the control (*p*<0.01). Regarding chemokine concentrations, the statistical analysis revealed a main effect of SUD on CXCL12 (*p*<0.01) and CCL11 (*p*<0.05) concentrations. The multiple comparisons test showed significantly lower CXCL12 concentrations in the alcohol group than in the cocaine (*p*<0.05) and control (*p*<0.01) groups, but also significantly lower CCL11 (*p*<0.05) in the cocaine group than in the control (*p*<0.05). In contrast, no main effects of SUD were detected on plasma concentrations of CCL2 and CX3CL1.

### Inflammatory mediators in relation to comorbid major depressive disorders

We investigated whether comorbid MDD was associated with alterations in the plasma concentrations of these inflammatory mediators in the two groups of SUD patients. A one-way ANCOVA was performed for the cocaine (CUD/non-MDD and CUD/MDD patients) and alcohol groups (AUD/non-MDD and AUD/MDD patients) controlling for sex, age, BMI, and antidepressant medication. Plasma concentrations and statistical analysis of inflammatory mediators according to MDD in the cocaine and alcohol groups are described in Table 3.

**Table 3:** Plasma concentrations of inflammatory mediators in the cocaine and alcohol groups according to comorbid depression.

VARIABLE	COCAINE GROUP (CUD) (n = 64)		ANCOVA (Statistics) ^(1)^			ALCOHOL GROUP (AUD) (n = 65)		ANCOVA (Statistics) ^(1)^		
	Non-MDD (n = 31)	MDD (n = 33)	F-value	df	*p*-value	Non-MDD (n = 27)	MDD (n = 38)	F-value	df	*p*-value
	Mean ± SD	Mean ± SD				Mean ± SD	Mean ± SD			
**TNF-α** (pg/mL)	8.00±5.11	9.80±5.58	0.992	1,57	0.319	10.27±5.97	9.67±5.03	0.077	1,59	0.782
**IL-1β** (pg/mL)	1.346±0.868	0.699±0.327	4.211	1,46	**0.046**	1.764±1.129	1.646±1.137	0.105	1,59	0.747
**CXCL12** (pg/mL)	287.9±60.34	252.5±41.04	5.222	1,58	**0.026**	240.1±77.69	241.8±110.3	0.053	1,59	0.819
**CCL2** (pg/mL)	38.70±9.303	43.36±12.89	0.853	1,58	0.360	39.01±16.28	34.89±16.14	1.568	1,59	0.215
**CCL11** (pg/mL)	126.5±54.00	171.10±98.14	4.778	1,58	**0.033**	128.8±37.10	111.9±35.3	2.899	1,59	0.094
**CX3CL1** (pg/mL)	1.987±1.666	2.342±1.252	5.807	1,55	**0.019**	1.839±1.090	2.212±1.298	1.742	1,59	0.192

^**(1)**^ Statistical analysis was conducted on the logarithmic transformed values to ensure statistical assumptions. Data were analyzed by ANCOVA controlling for age, sex, BMI, and antidepressant medication

Missing participants: TNF-α (n = 1, CUD/MDD); IL-1β (n = 7, CUD/non-MDD; n = 5, CUD/MDD); CX3CL1 (n = 1, CUD/non-MDD; n = 2, CUD/MDD)

Abbreviations: ANCOVA = analysis of covariance; AUD = alcohol use disorders; CUD = cocaine use disorders; df = degree of freedom; MDD = major depressive disorder

One-way ANCOVA showed a main effect of MDD on the plasma concentrations of IL-1β (*p*<0.05), CXCL12 (*p*<0.05), CCL11 (*p*<0.05), and CX3CL1 (*p*<0.05) in the cocaine group, but no significant effects on any of these inflammatory mediators in the alcohol one. CUD/MDD patients showed significantly lower IL-1β and CXCL12 concentrations than CUD/non-MDD patients, and significantly higher CCL11 and CX3CL1 concentrations than CUD/non-MDD patients.

### Inflammatory mediators in relation to primary and substance-induced major depressive disorders

SUD patients who were diagnosed with MDD were divided into primary and substance-induced MDD according to DSM-IV-TR criteria. We investigated the impact of the type of comorbid MDD in CUD and AUD patients using one-way ANCOVA for the cocaine (CUD/non-MDD, CUD/primary MDD, and CUD/cocaine-induced MDD patients) and alcohol groups (AUD/non-MDD, AUD/primary MDD, and AUD/cocaine-induced MDD patients), controlling for sex, age, BMI, and antidepressant medication. Plasma concentrations and statistical analysis of inflammatory mediators according to type of MDD in the cocaine and alcohol groups are described in Table 4.

**Table 4:** Plasma concentrations of inflammatory mediators in the cocaine and alcohol groups according to the type of depression.

VARIABLE	COCAINE GROUP (CUD) (n = 64)			ANCOVA (Statistics) ^(1)^			ALCOHOL GROUP (AUD) (n = 65)			ANCOVA (Statistics) ^(1)^		
	Non-MDD (n = 31)	Primary MDD (n = 18)	Cocaine-Induced-MDD (n = 15)	F-value	df	*p*-value	Non-MDD (n = 27)	Primary MDD (n = 16)	Alcohol-Induced-MDD (n = 22)	F-value	df	*p*-value
	Mean ± SD	Mean ± SD	Mean ± SD				Mean ± SD	Mean ± SD	Mean ± SD			
**TNF-α** (pg/mL)	8.00±5.11	11.63±6.38	7.45±3.25	0.751	2,56	0.477	10.27±5.97	9.62±5.17	9.70±5.05	0.102	2,58	0.875
**IL-1β** (pg/mL)	1.346±0.868	0.660±0.389	0.760±0.202	2.411	2,45	0.101	1.764±1.129	1.343±0.692	1.867±1.347	0.217	2,58	0.806
**CXCL12** (pg/mL)	287.9±60.34	265.5±42.56	236.9±34.26 ^**a**^	4.453	2,57	**0.016**	240.1±77.69	248.7±66.69	236.8±134.8	0.415	2,58	0.662
**CCL2** (pg/mL)	38.70±9.303	45.40±14.15	40.91±11.16	0.501	2,57	0.609	39.01±16.28	30.96±13.71	37.74±17.45	1.444	2,58	0.244
**CCL11** (pg/mL)	126.5±54.00	211.7±111.4 ^**aa**^	124.3±49.9 ^**bb**^	6.445	2,57	**0.003**	128.8±37.10	115.4±39.4	109.3±32.8	1.401	2,58	0.255
**CX3CL1** (pg/mL)	1.987±1.666	2.272±1.195	2.439±1.371	0.134	2,54	0.875	1.839±1.090	2.116±1.387	2.283±1.259	0.864	2,58	0.427

^**(1)**^ Statistical analysis was conducted on the logarithmic transformed values to ensure statistical assumptions. Data were analyzed by ANCOVA controlling for age, sex, BMI, and antidepressant medication.

^**(a)**^ *p*<0.05 and

^**(aa)**^ *p*<0.01 denote significant differences versus cocaine non-MDD group after *post hoc* comparisons

^**(bb)**^ *p*<0.01 denotes significant differences versus CUD primary MDD group after *post hoc* comparisons

Missing participants: TNF-α (n = 1, CUD/cocaine-induced MDD); IL-1β (n = 7, CUD/non-MDD; n = 1, CUD/primary MDD; n = 4, CUD/cocaine-induced MDD); CX3CL1 (n = 1, CUD/non-MDD; n = 2, CUD/cocaine-induced MDD)

Abbreviations: ANCOVA = analysis of covariance; AUD = alcohol use disorders; CUD = cocaine use disorders; df = degree of freedom; MDD = major depressive disorders

In a similar manner to previous results with comorbid MDD diagnosis, one-way ANCOVA revealed a main effect of the type of MDD on the plasma concentrations of CXCL12 (*p*<0.05) and CCL11 (*p*<0.01) in the cocaine group, but not in the alcohol one. The multiple comparisons test showed that CXCL12 concentrations were significantly lower in CUD/cocaine-induced MDD patients than in CUD/non-MDD patients (*p*<0.05). The *post hoc* test also revealed that CCL11 concentrations were significantly higher in CUD/primary MDD patients than in CUD/non-MDD (*p*<0.01) and CUD/cocaine-induced MDD (*p*<0.01) patients.

### Inflammatory mediators in cocaine use disorders as potential biomarkers of comorbid major depressive disorders

Logistic regression models were generated to evaluate whether these cytokine and chemokine concentrations could serve as explanatory variables in order to discriminate between primary MDD and substance induced-MDD in the cocaine group. The regression analyses included additional variables (age, sex, and BMI) using different approaches and the resulting models are represented in Table 5.

**Table 5:** Binary logistic regression models for predicting cocaine-induced depression through plasma concentrations of inflammatory mediators, sex, age, and BMI.

MODEL	VARIABLE	B	SEM	W	df	*p* value	Exp(B)	95% CI for Exp(B)	
								Lower	Upper
**LOGISTIC REGRESSION FULL MODEL 1** **Method: Enter**	**TNF-α**	-0.204	0.144	2.005	1	0.157	0.816	0.616	1.081
	**IL-1β**	2.809	2.493	1.270	1	0.260	16.597	0.125	2198
	**CXCL12**	-0.021	0.017	1.401	1	0.237	0.980	0.947	1.014
	**CCL2**	0.070	0.073	0.921	1	0.337	1.073	0.930	1.238
	**CCL11**	-0.017	0.012	1.890	1	0.169	0.983	0.959	1.007
	**CX3CL1**	0.308	0.602	0.262	1	0.609	1.361	0.418	4.426
	**Age**	-0.019	0.106	0.033	1	0.856	0.981	0.797	1.208
	**Sex**	2.788	1.503	3.443	1	0.064	16.25	0.855	308.8
	**BMI**	-0.084	0.170	0.243	1	0.622	0.920	0.659	1.283
	**Constant**	5.369	5.991	0.803	1	0.370	214.7	-	-
**LOGISTIC REGRESSION MODEL 2** **Method: Backward Stepwise**	**CXCL12**	-0.022	0.013	2.829	1	0.093	0.978	0.954	1.004
	**CCL11**	-0.015	0.007	4.567	1	0.033	0.985	0.971	0.999
	**Sex**	2.037	0.976	4.354	1	0.037	7.669	1.132	51.973
	**Constant**	6.610	3.506	3.555	1	0.059	742.329		

Abbreviations: B = coefficient; SEM = standard error; W = Wald test; df = degrees of Freedom; CI = confidence interval

**Fig 1:**
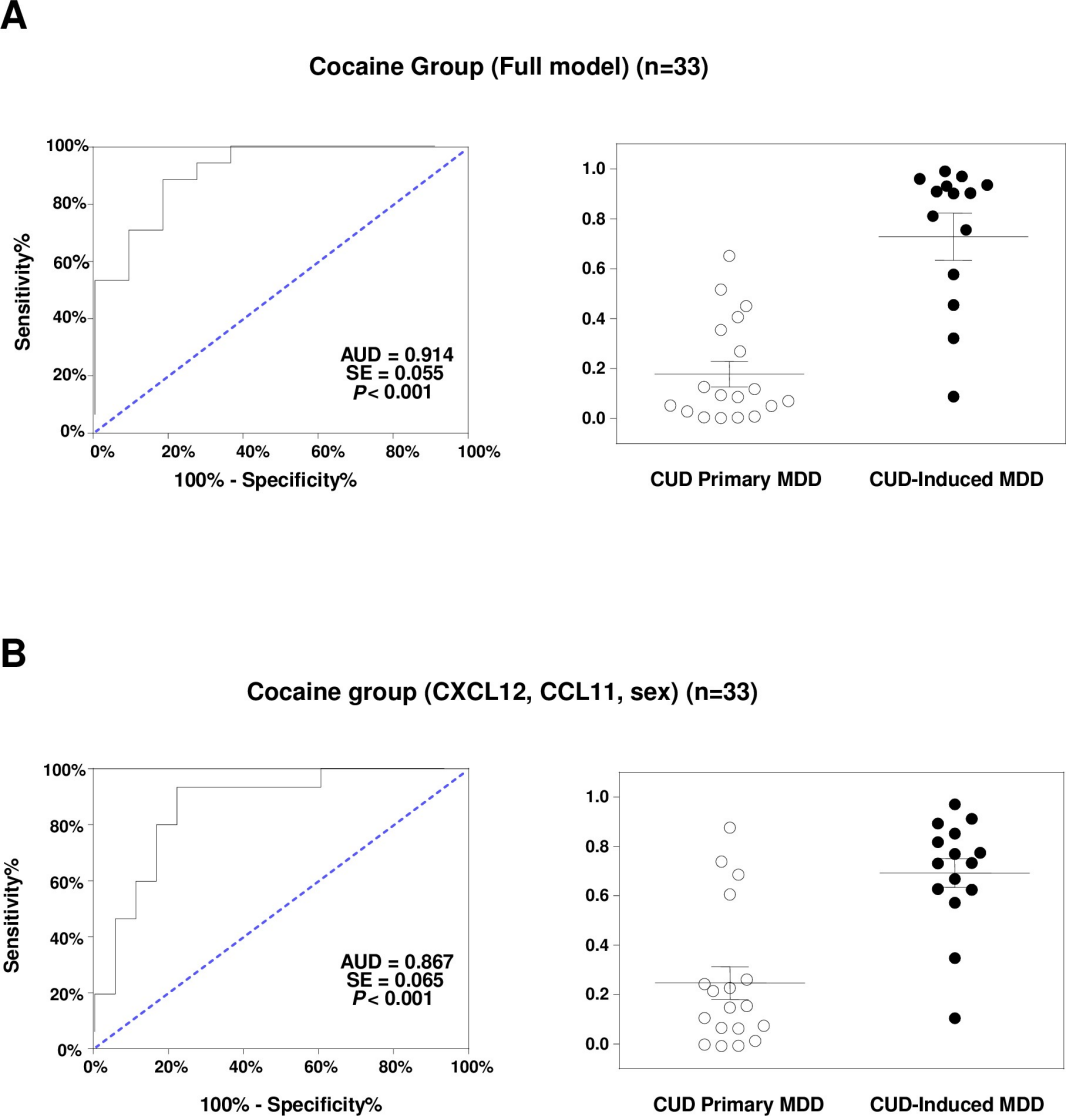
ROC analysis and scatter dots for two logistic models to distinguish primary MDD from cocaine-induced MDD in the cocaine group. (A) ROC analysis of a full model: TNF-α, IL-1β, CXCL12, CCL2, CCL11 CX3CL1, sex, age and BMI; and a scatter plot for the predictive probabilities. (B) ROC analysis of an adjusted model: CXCL12, CCL11 and sex; and a scatter plot for the predictive probabilities. Lines on the scatter plots are means and SD.

The full model included plasma concentrations of TNF-α, IL-1β, CXCL12, CCL2, CCL11, and CX3CL1, and age, sex and BMI as independent variables in the logistic regression analysis in the cocaine group. As depicted in Fig 1A, the ROC curve analysis showed an AUC = 0.914 (*p*<0.001), which indicates a high discriminative power. A representative cut-off value was 0.452 [sensitivity 88.24 (95%CI = 63.56–98.54) and specificity 81.82 (95% CI = 48.22–97.72)]. Regarding the scatter plot of the predictive probabilities for the type of MDD, their means were significantly different between primary MDD and cocaine-induced MDD patients (U = 16.00, *p*<0.001).

To identify the most discriminative variables, a backward stepwise approach was used to generate a logistic regression model in the cocaine group. This model was restricted to 3 explanatory variables in the equation: CXCL12 and CCL11 concentrations and sex. As depicted in Fig 1B, the ROC analysis showed an AUC = 0.867 (*p*<0.001), maintaining a high discriminative power. A representative cut-off value was 0.311 [sensitivity 77.78 (95%CI = 52.36–93.59) and specificity 93.33 (95% CI = 68.05–99.83)]. The predictive probabilities for the type of MDD also differed between primary MDD and cocaine-induced MDD patients (U = 36.00, *p*<0.001).

## Discussion

The main findings of this observational study are as follows: (a) There were differences in the plasma concentrations of some inflammatory mediators between CUD (TNF-α, IL-1β and CCL11) and AUD (IL-1β and CXCL12) patients when compared with control subjects; (b) CUD patients diagnosed with comorbid MDD showed significant differences in plasma concentrations of IL-1β, CXCL12, CCL11 and CX3CL1. In contrast, there were no differences in the alcohol group; (c) Plasma CCL11 concentrations were found to be higher in CUD patients with primary MDD than those with cocaine-induced MDD.

Evidence has revealed that the immune system participates in the mechanisms involved in long-term adaptation and neurotoxicity associated with the substance use [35]. As consequence of microglia activation, blood-brain-barrier disruption and neuroinflammation, proinflammatory mediators such as cytokines and chemokines are altered in the brain [36–37]. However, these changes are not restricted to the Central Nervous System, and alterations in these inflammatory signals have been also found in the blood of SUD patients and proposed as biomarkers of such disorders [38]. Thus, our results revealed that abstinent CUD patients had lower concentrations of TNF-α and CCL11, and higher concentrations of IL-1β relative to controls. Accordingly, a previous study in abstinent patients with a pathological use of cocaine reported lower concentrations of TNF-α than controls and elevated concentrations of IL-1β in those patients with a high number of DMS-IV-TR criteria for CUD [11]. These peripheral alterations of relevant cytokines and CCL11 in the plasma of cocaine users could be associated with changes in the expression of immune blood cells. In fact, acute cocaine administration in rats produces increased levels of neutrophils and decreased levels of leukocytes and lymphocytes [39]. Interestingly, a study with cocaine-dependent volunteers reported a decrease in the capacity of monocytes to express TNF-α and IL-6 compared with controls [40]. Unlike the archetypal proinflammatory cytokines TNF-α and IL-1β, there is no literature linking cocaine exposure with changes in plasma levels of CCL11. In contrast, a cross-sectional study has suggested that current cannabis use is linked to increased plasma levels of CCL11 [41]. Regarding AUD patients, significant alterations in the plasma were observed for IL-1β and CXCL12. Similar to CUD patients, high concentrations of IL-1β were found in AUD patients, which has been associated with an altered pattern of production of inflammatory cytokines in peripheral blood monocytes from chronic alcohol patients [42]. In addition to IL-1β, a specific decrease in CXCL12 concentrations in AUD patients was found, which is in agreement with a previous study from our group examining plasma chemokines in abstinent AUD patients in treatment [30].

Among mood disorders, MDD is the comorbid psychiatric disorder more prevalent in SUD patients [7]. Because cytokines play a role in neuronal integrity, neurogenesis and synaptic remodeling, growing evidence suggests that inflammatory mediators are involved in the development of MDD [43]. Moreover, numerous studies have reported changes in circulating proinflammatory cytokines in patients with MDD [44]. In the present study, we explored circulating cytokines and chemokines in SUD patients diagnosed with MDD. Notably, while the diagnosis of comorbid MDD was associated with alterations in plasma concentrations of some cytokines and chemokines in the CUD group, no differences were found in the AUD group. Thus, CUD patients with comorbid MDD showed lower concentrations of IL-1β and CXCL12, and higher concentrations of CX3CL1 and CCL11 as compared with than those CUD patients without this condition. These results suggest that the type of SUD could have a prominent effect on the detection of alterations in inflammatory mediators associated with comorbid MDD. In addition to the type of substance, differences in other clinical and sociodemographic variables between both groups could be affecting the expression of circulating inflammatory mediators, for example age and sex. Previously, we reported that plasma concentrations of IL-1β, CXCL12 and CX3CL1 are positively associated with the number of DSM-IV-TR criteria for cocaine abuse and dependence. However, although cocaine users with severe CUD displayed an elevated prevalence of mood disorders, no differences in the plasma concentrations of these inflammatory mediators were reported [11]. In contrast, another recent study in the same cohort with CUD patients reported no changes of CCL11 concentrations relative to addiction-related variables or diagnosis of psychiatric comorbidity [13]. It is important to point out that in both studies we explored the association of these inflammatory mediators with psychiatric comorbidity at a level more general (mood disorders [11] or psychiatric DSM-IV-TR Axis I disorders and personality disorders [13]). While we observed lower plasma concentrations of IL-1β and CXCL12 in CUD patients with comorbid MDD, other studies conducted in psychiatric patients with no interference of substances have showed opposite results. For example, while increased IL-1β concentrations have been reported in depressed individuals compared with controls [45], another clinical study showed increased plasma levels of CXCL12 in MDD patients with different severities of depressive conditions compared with a control group [46]. These data suggest that the co-occurrence of CUD and MDD could prevent increased plasma concentrations of IL-1β and/or CXCL12 concentrations when CUD or MDD are not comorbid. Unlike IL-1β and CXCL12, chemokines CX3CL1 and CCL11 were found increased in CUD patients with MDD. Accordingly, transient elevated serum levels of CX3CL1 have been described in colorectal cancer patients with anxiety and depression [47]. Regarding CCL11, considerable evidence exists on the role of this chemokine in the progression of neurodegenerative diseases (e.g., schizophrenia or dementia patients) [22, 48], but contradictory results have been reported in relation to MDD. Thus, while changes in CCL11 are observed in women with recurrent MDD with suicidal ideation [24] and in young adults with mood disorders [49], another study in elderly patients with MDD showed no changes [50].

Because there was a strong association between alterations in the plasma expression of certain inflammatory mediators and comorbid MDD, we extended our study in CUD patients diagnosed with primary and cocaine-induced MDD. In fact, the differentiation between primary and substance-induced MDD in dual pathology is a field of study to be explored, and a recent research has showed that platelet IRAS/nischarim (I_1_-Imidazoline receptor) could discriminate between primary MDD and cocaine-induced MDD in CUD patients [51]. In our study, we have observed differences in the plasma expression of CCL11 and CXCL12 according to the type of MDD. Thus, CCL11 was increased in CUD patients with primary MDD relative to those with cocaine-induced MDD and non-comorbid patients. In the case of CXCL12, CUD patients with cocaine-induced MDD showed decreased CXCL12 concentrations relative to those with primary MDD and non-comorbid patients. Then, the ROC analysis of regression models confirmed that CXCL12 and CCL11, in combination with sex, had a strong discriminative power to discriminate between both types of MDD.

### Limitations and strengths

Although our findings support the clinical importance of a differential diagnosis for MDD in CUD and AUD, we are aware of important limitations. First, there is a growing number of additional social, environmental and addiction-related variables (covariates) that could influence our data. Second, because the participants were under current treatment for SUD at the moment of evaluation, we cannot exclude an influence of other psychiatric medications (e.g., anxiolytics and antipsychotics) on these inflammatory signals. Third, the impact of sex/gender in the expression of inflammatory mediators is critical and, therefore, it is required a larger sample size and a better characterization including other variables (e.g., sex hormones). Fourth, a cross-validation of the proposed discriminative models is necessary to ensure reliability and stability of the estimations. Fifth, the inclusion of patients diagnosed with MDD and no history of SUD and healthy controls are needed for future research, even though these psychiatric individuals have been previously described in the literature [45].

The strengths of the study are that we measured inflammatory mediators in a well-phenotyped cohort of subjects characterized by a marked presence of comorbid depressive disorders. Furthermore, we employed a diagnostic procedure with good test-retest reliability, validity, and inner rater reliability in the differentiation between primary and substance-induced MDD [32].

## Conclusions

Patients with dual depression have worse clinical evolution and worse therapeutic response. Namely, the efficacy of antidepressant medication and/or psychological therapies in patients with AUD or CUD and comorbid MDD can be influenced by the type of depression (i.e. primary and substance-induced MDD) (DSM-IV-TR). However, there are no biological substrates that can reveal the difference between both mental conditions for a better clinical management of these patients. The present study represents an attempt at the identification of inflammatory biomarkers for the differentiation between primary and substance-induced disorders. Although our findings indicate that plasma concentrations of chemokine CCL11 could act as a potential biological marker to differentiate between primary and substance-induced MDD in CUD patients, further clinical research is necessary to confirm the role of inflammatory mediators in dual depression.

## Supporting information

S1 File Dataset. Dataset includes: Sex (man/woman); SUD (no/yes), SUD type (no/AUD/CUD), MDD (no/yes), MDD type (no/primary MDD/induced MDD), and concentrations (pg/mL) of TNF-α, IL-β, CXCL12, CCL2, CCL11 and CX3CL1.
(PDF)
