# Supplementary material for: Inflammatory mediators and dual depression: Potential biomarkers in plasma of primary and substance-induced major depression in cocaine and alcohol use disorders
Source: PLoS One. 2019 Mar 14;14(3):e0213791. doi: 10.1371/journal.pone.0213791 (PMC6417778; doi:10.1371/journal.pone.0213791)
Supplement: S1 File — Dataset includes: Sex (man/woman); SUD (no/yes), SUD type (no/AUD/CUD), MDD (no/yes), MDD type (no/primary MDD/induced MDD), and concentrations (pg/mL) of TNF-α, IL-β, CXCL12, CCL2, CCL11 and CX3CL1. (PDF) [file pone.0213791.s001.pdf]

CODE	AGE<br>years	BMI<br>kg/m2	SEX<br>1=male<br>2=female	SUD<br>0=No<br>1=Yes	SUD TYPE<br>0=No<br>1=CUD<br>2=AUD	MDD<br>0=No<br>1=Yes	MDD TYPE<br>0=No<br>1=Primary MDD<br>2=Induced MDD	TNFa<br>pg/mL	IL1b<br>pg/mL	CXCL12<br>pg/mL	CCL2<br>pg/mL	CCL11<br>pg/mL	CX3CL1<br>pg/mL
	25	28.7	1	0	0	0	0	13.6	0.39	260.25	44.68	156.25	5.65
	36	28.9	1	0	0	0	0	9.9	1.05	270.59	48.19	172.88	4.84
	38	26.5	1	0	0	0	0	16	0.65	227.19	60.93	141.37	5.41
	39	27.7	1	0	0	0	0	9.5	0.68	303.81	82.98	392	2.94
	30	22.8	2	0	0	0	0	11.9	0.62	276.31	85.35	306.19	2.96
	30	24.3	2	0	0	0	0	14.4	0.45	305.29	48.51	186.86	1.43
	29	19.8	1	0	0	0	0	15.2	0.92	299.9	48.37	182.77	6.52
	29	28	2	0	0	0	0	20.4	0.95	281.4	60.92	170.5	1.8
	42	20.4	2	0	0	0	0	15.2	0.48	314.86	50.96	134.65	4.23
	32	23.8	1	0	0	0	0	11.5	0.27	303.81	47.15	161.31	0.81
	41	26.4	2	0	0	0	0	11.9	1.18	310.23	56.36	286.99	2.6
	31	27.2	2	0	0	0	0	15.2	0.27	279.15	54.51	132.23	2.28
	37	26.4	2	0	0	0	0	21.6	1.05	291.7	57.41	206.41	4.01
	29	25	2	0	0	0	0	10.3	0.45	252.08	37.97	143.27	2.48
	32	25.6	2	0	0	0	0	6.1	0.51	302.74	39.66	107.59	2.59
	33	25.7	2	0	0	0	0	20	0.51	307.83	57.93	193.61	3.13
	34	26.6	2	0	0	0	0	7	0.48	253.58	48.07	151.02	3.07
	23	23	1	0	0	0	0	17.2	0.51	288.66	37.86	140.34	3.46
	33	20.9	1	0	0	0	0	13.6	0.79	300.71	67.37	195.19	6.87
	33	24.3	1	0	0	0	0	5.3	3.93	281.82	57.81	174.04	4.24
	36	22.5	1	0	0	0	0	12.7	0.73	348.96	44.99	87.67	3.51
	35	21.6	1	0	0	0	0	18	0.62	275.74	40.63	174.65	3.2
	34	20.06	2	0	0	0	0	6.5	0.69	203.59	17.63	126.69	3.63
	33	23.05	1	0	0	0	0	5	4.75	396.22	15.01	174.82	3.01
	43	25.71	1	0	0	0	0	16.8	0.51	323.46	28.85	205.75	2.31
	35	24.39	1	0	0	0	0	9.2	0.6	251.78	21.89	178.7	0.87
	30	30.12	2	0	0	0	0	18.4	0.82	284.85	42.72	121.48	2.46
	30	21.77	2	0	0	0	0			293.34	22.18	36.33	2.02
	38	25.68	1	0	0	0	0	1.6	0.65	219.61	26.34	95.09	1.35
	37	24.8	1	0	0	0	0	5	0.86	265.49	29.51	114.3	1.45
	37	27.78	1	0	0	0	0	5	0.86	270.15	23.64	71.5	1.56
	39	21.04	1	0	0	0	0	16.1	1.11	335.87	14.99	193.65	1.45

43	23.89	1	0	0	0	0	13	0.23	267.82	20.3	106.87	1.44
38	27.13	1	0	0	0	0	4.5		249.74	16.73	118.13	2.03
38	29.32	1	0	0	0	0	3.5		248.34	22.87	114.11	0.67
28	22.09	1	0	0	0	0	9.4	0.56	250.06	30.24	203.24	2.96
37	26.83	1	0	0	0	0	3.5		214.35	21.62	65.71	1.61
27	23.6	1	0	0	0	0	13.6	0.3	296.43	29.15	184.54	3.16
28	20.6	2	0	0	0	0	19.9	0.49	246.91	28.9	64.27	4.4
30	26.23	1	0	0	0	0	10.2	0.49	329.6	28.33	108.55	3.85
27	24.3	1	0	0	0	0	11.6	0.38	287.36	19.77	109.18	1.88
39	27.76	1	0	0	0	0	12	0.57	251.01	38.64	185.2	1.21
30	24.05	1	0	0	0	0	7.7	0.08	292.88	27.91	132.92	1.22
42	20.53	1	0	0	0	0	10.7	0	327.53	30.46	148.3	1.7
40	34.34	1	0	0	0	0	15	0.81	350.82	26.26	104.9	2.38
39	29.71	1	0	0	0	0	5.9	0	328.32	23.28	104.36	0.24
35	26.04	2	0	0	0	0	12.9	1.41	263.74	30.09	269.74	2.57
48	29.39	2	0	0	0	0	5.2	0.66	236.38	30.8	145.96	2.86
25	23.44	2	0	0	0	0	12.2	0.32	254.99	31.01	67.89	1.74
22	22.92	1	0	0	0	0	7	0.61	293.08	24.82	149.73	2.02
43	19.72	2	0	0	0	0	11.6	0.3	211.87	27.81	205.68	2.35
49	19.82	2	0	0	0	0	12.3	0.53	264.24	21.16	81	2.15
40	23.73	2	0	0	0	0	9.8	0.98	248.61	20.51	156.16	1.52
25	37.04	2	0	0	0	0	6.8	0.34	260.07	22.32	98.69	0.52
24	21.91	1	0	0	0	0	8	0.1	373.02	12.75	152.28	1.85
35	22.86	2	0	0	0	0	10.5	0.7	351.61	19.18	123.67	2.87
40	21.97	2	0	0	0	0	20.7	1	286.97	27.42	122.31	1.79
37	29.76	2	0	0	0	0	7.9	0.28	311.68	24.03	105.73	2.12
42	25.33	2	0	0	0	0	17	1.28	277.89	21.47	124.74	3.34
44	20.32	2	0	0	0	0	12.7	0.45	441.97	30.08	175.81	1.21
44	25.51	2	0	0	0	0	5.2	0.4	358.04	36.24	83.67	3.45
37	22.43	2	0	0	0	0	10	0.4	249.31	22.71	77.67	2.31
35	20.7	2	1	1	1	2	8.6	0.68	270.73	34.29	177.6	2.33
23	19.5	1	1	1	1	2	7.4	0.62	237.27	57.88	190.37	3.94
26	27.8	2	1	1	1	2	1.9	1.09	207.93	32.36	113.84	3.36
44	24.8	2	1	1	1	2	10.4	0.63	196.13	45.13	115.7	3.44
35	26.3	1	1	1	1	2	4.4	0.59	264.59	45.8	138.88	2.51

31	24.1	2	1	1	1	2	6.3	0.53	188.18	55.71	96.32	4.28
48	24.9	1	1	1	1	2	8.3	1.03	186.35	42.9	73.7	1.12
59	29.9	1	1	1	1	2	4.7	0.89	195.48	35.54	146.07	3.72
39	33.6	1	1	1	1	2	12.3	0.56	234.83	49.96	148.79	0.08
32	34.34	1	1	1	1	2	7.4		273.94	52.37	91.57	0.9
31	28.38	1	1	1	1	2	12.8		256.51	39.38	99.24	
27	21.86	1	1	1	1	2			252.43	17.91	51.34	
31	27.17	1	1	1	1	2	2.7	0.97	292.14	27.33	79.83	0.73
33	24.22	1	1	1	1	2	8.7		243.67	45.8	101.35	3.25
41	24.9	1	1	1	1	2	8.4	0.77	253.47	31.3	239.98	2.04
45	28.4	2	1	1	1	1	20	0.48	272.89	46.6	174.22	2.94
45	21.48	1	1	1	1	1	18.4	0.68	271.31	66.84	285.25	4.89
46	34.2	2	1	1	1	1	6	0.62	269.58	37.09	122.72	2.85
55	33.75	2	1	1	1	1	8.6	0.1	215.37	41.52	71.64	2.53
35	31.82	1	1	1	1	1	8	0.45	281.96	33.45	92.66	1.25
24	31.3	2	1	1	1	1	13.2	0.13	283.09	57.88	133.24	3.71
42	23	2	1	1	1	1	8.2	0.89	336.68	72.89	265.15	1.62
51	25.6	2	1	1	1	1	11.1	0.79	263.19	62.13	464.98	1.77
39	26.6	2	1	1	1	1	15.2	0.7	292.25	50.38	169.62	1.73
47	27.5	2	1	1	1	1	8.6	0.68	300.44	47.31	396.39	3.95
37	23.5	2	1	1	1	1	5.3	0.84	216.36	36.91	287.32	0.57
56	40.9	2	1	1	1	1	7	0.81	244.51	59.48	244.85	3.36
49	21.6	1	1	1	1	1	15.6	0.36	235.87	45.05	297.15	1.77
42	23.5	2	1	1	1	1	26.3	0.89	295.68	36.16	254.43	0.63
42	24.92	2	1	1	1	1	0.1	0.64	237.76	41.33	172.24	2.42
22	18.66	2	1	1	1	1	16.3	1.83	313.61	18.24	48.3	1.34
23	21.22	1	1	1	1	1	15		292.5	35.7	116.85	2.58
36	22.53	1	1	1	1	1	6.5	0.33	155.39	28.27	213.55	0.98
29	25.2	2	1	1	0	0	15	1.37	311.38	41.93	69.85	4.63
41	23.2	1	1	1	0	0	6.1	1.43	260.77	46.97	142.76	0.98
25	22.5	1	1	1	0	0	3.3	0.92	248.13	64.97	145.06	0.95
34	22.8	1	1	1	0	0	6.5	1	268.79	44.91	96.95	3.65
26	22.7	1	1	1	0	0	6.1	0.81	262.04	47.97	152.65	2.48
39	25.9	1	1	1	0	0	5.8	1.14	258.47	57.59	147.74	3.36
41	31.7	2	1	1	0	0	8.6	1.19	187.14	27.54	41.94	2.21

43	26.2	1	1	1	0	0	8.3	0.75	288.51	35.28	67.71	2.64
32	20.3	1	1	1	0	0	3.7	1.03	241.23	43.69	237.23	0.29
43	23.3	1	1	1	0	0	8.3	1.72	249.28	41.31	120.05	2.14
46	30.67	1	1	1	0	0	2	0.97	261.46	39.15	80.81	1.5
40	25.03	1	1	1	0	0	12.5	2.45	161.5	17.44	72.47	0.34
38	25.08	1	1	1	0	0	7.8	2.78	248.46	37.92	124.75	1.63
29	24.38	1	1	1	0	0	20	4.07	322.98	37.38	135.44	2.84
25	25.04	2	1	1	0	0	7.8		332.76	51.8	93.58	3.81
37	28.06	1	1	1	0	0	23.5	2.26	323.93	27.01	181.76	1.86
39	28.08	1	1	1	0	0	8	0.97	371.06	32.21	86.18	0.32
35	22.79	1	1	1	0	0	11.4		400.22	45.26	123.04	3.09
38	26.25	1	1	1	0	0	8.7		365.95	32.4	95.59	0.53
37	18.73	2	1	1	0	0	4.9	0.79	338.58	32.73	44.68	1.99
38	28.34	1	1	1	0	0	12.5	1.02	235.93	26.89	251.46	2.31
38	22.65	1	1	1	0	0	0.1	1.02	388.97	30.97	81.5	1.45
36	23.12	1	1	1	0	0	4.4	1.83	303.67	34.94	145.24	1.09
43	25.22	1	1	1	0	0	12.3		245.17	39.5	140.22	1.04
24	27.45	1	1	1	0	0	4	0.13	218.34	34.89	221.65	1.06
43	21.33	2	1	1	0	0	6.5	0.13	231.98	35.97	177.72	
27	19.95	1	1	1	0	0	5.2		287.85	36.46	118.1	2.56
45	25.14	1	1	1	0	0	11.1	1.78	345.42	35.4	148.43	2.75
43	27.72	1	1	1	0	0	0.1	0.75	279.2	39.45	175.83	1.51
43	24.69	1	1	1	0	0	6.9		403.29	39.85	154.91	0.79
35	20.31	2	1	1	0	0	6.5		283.32	39.84	45.38	3.81
42	30.6	1	1	2	1	1	9.7	1.19	211.04	50.79	145.22	1.41
58	30.1	1	1	2	1	1	4	1.89	239.06	45.14	202.61	2.8
45	28.7	2	1	2	1	1	5.1	1.09	237.65	51.36	140.54	5.29
52	31	1	1	2	1	1	5.4	0.43	273.25	47.83	155.76	1.27
44	26.2	2	1	2	1	1	8.8	0.1	360.77	23.53	68.12	0.92
48	31.7	1	1	2	1	1	5.8	0.75	281.13	30.93	133.69	2.91
40	28.1	2	1	2	1	2	4.4	0.35	155.55	28.98	127.72	3.36
41	25.4	2	1	2	1	2	4.4	0.63	183.21	61.72	130.7	3.37
46	28.1	1	1	2	1	2	7.2	1.14	191.97	40.86	26.95	3.38
38	31.4	1	1	2	1	2	5.9	0.69	191.05	31.57	97.76	3.74
34	21.2	1	1	2	1	2	1	1.48	226.75	41.8	133.95	5.48

51	21.8	1	1	2	1	2	6.3	0.75	237.65	38.21	77.74	2.49
53	22	2	1	2	1	2	1.9	0.24	246.73	49.42	128.42	2.37
45	27	1	1	2	0	0	0.5	0.43	193.01	39.7	133.02	2.29
45	23.6	1	1	2	0	0	6.1	0.19	217.49	31.12	187.68	1.76
39	25.4	1	1	2	0	0	14.8	1.98	223.15	53.84	125.97	2.78
53	29.3	2	1	2	0	0	6.1	0.1	253.88	43.9	80.93	2.91
37	27.3	1	1	2	0	0	7.5	0.5	236.5	45.39	157.34	0.28
51	25.9	1	1	2	0	0	7.5	1.14	222.12	56.68	138.13	5.64
43	27.7	1	1	2	0	0	3	0.63	234.96	48.06	201.17	2.55
41	25.8	1	1	2	0	0	9	0.56	238.03	31.59	111.09	0.82
42	19.38	2	1	2	0	0	6.61	1.84	157.78	30.43	77.39	1.34
46	25.5	1	1	2	1	2	6.94	1.22	167.54	31.84	144.42	0.89
49	20.7	2	1	2	1	2	15.4	5.31	196.05	34.11	80.74	3.86
61	19.03	2	1	2	1	2	15.22	2.81	152.83	29.78	76.04	2.05
56	20.37	1	1	2	1	2	7.41	1.12	141.76	10.84	121.11	0.81
60	27.5	1	1	2	1	2	12.2	2.42	784.53	13.97	142.47	1.76
49	34.7	1	1	2	1	1	10.49	1.94	259.68	17.89	117.59	1.66
41	26.67	1	1	2	0	0	10.49	3.22	184.14	29.24	75.36	2.34
61	22	1	1	2	1	1	3.14	0.81	191.52	12.43	131.1	1.07
45	28.91	1	1	2	1	1	12.46	1.88	226.86	27.78	126.88	1.93
38	25.28	2	1	2	1	2	15.51	3.16	238.95	29.53	107.34	2.3
49	33.77	2	1	2	1	1	10.78	1.83	203.21	16.61	56.05	1.46
51	26.8	1	1	2	0	0	10.2	2.19	214.14	29.18	104.36	1.59
45	20.08	1	1	2	1	1	12.82	1.82	198.93	34.27	102.78	1.2
59	29.01	2	1	2	1	2	5.21	0.93	138.42	28.17	127.13	0.68
37	18.9	1	1	2	1	1	11.58	1.59	199.67	21.44	56.68	1.28
44	24.77	1	1	2	1	1	12.82	1.69	414.24	22.93	108.27	4.06
47	23.15	2	1	2	1	2	13.55	2.2	197.32	22.02	60.44	1.6
49	28.08	1	1	2	1	2	8.99	1.45	197.44	31.99	100.98	1.05
33	26.03	1	1	2	1	2	9.03	3.98	310.52	54.28	108.66	2.89
47	23.23	2	1	2	1	2	14.2	4.42	387.27	87.12	66.61	3.21
42	31.14	1	1	2	0	0	8.81	1.2	144.93	20.3	119.9	0.88
58	25.1	1	1	2	0	0	10.93	1.36	193.1	41.36	136.03	0.99
31	25.15	1	1	2	1	2	10.56	1.5	176.08	19.16	118.26	1.09
51	25.3	2	1	2	1	1	7.93	1.18	172.79	15.32	88.45	0.97

42	29.14	2	1	2	0	0	10.18	1.92	159.44	13.59	135.24	1.4
49	33.06	2	1	2	0	0	13.21	1.55	228.64	44.25	113.54	1.13
60	23.05	1	1	2	0	0	7.27	1.27	225	78.14	155.17	0.93
48	31.18	1	1	2	0	0	14.24	1.63	333.06	52.53	141.21	1.18
43	27.97	2	1	2	1	1	8.21	0.58	196.53	28.29	78.68	1.11
51	25.01	2	1	2	0	0	17.35	2.2	236.37	31.08	172.17	1.6
58	27.12	2	1	2	0	0	6.46	1.45	232.17	31.68	104.67	1.06
39	29.05	1	1	2	0	0	5.25	1.84	325.67	19.62	83.86	1.34
44	19.81	1	1	2	0	0	10.85	2.85	363.91	50.83	120.15	2.08
52	19.32	1	1	2	0	0	10.58	4.73	269.49	54.15	120.14	3.44
34	20.83	1	1	2	0	0	28.55	4.39	536.22	76.5	97.07	3.19
48	29.6	1	1	2	0	0	21.89	1.52	208.35	27.5	109.4	1.11
44	28.31	1	1	2	1	1	24.91	2.71	313.28	48.88	134.17	4.49
48	32.6	1	1	2	1	2	19.23	1.8	219.96	48.19	132.28	1.31
43	26.49	2	1	2	1	2	13.26	2.09	251.13	60.85	159.56	1.52
56	24.91	1	1	2	1	2	15.49	1.37	217.46	35.84	136.17	0.99
51	21.01	2	1	2	0	0	11.53	2.61	205.61	28.66	105.99	1.9
45	21.89	1	1	2	0	0	10.29	2.13	253.64	20.59	146.05	1.55
54	20.82	1	1	2	0	0	7.99	2.18	191.73	23.4	225.52	1.58
